# The Involvement of Herpesviruses in the Pathogenesis of Thoracic Aortic Aneurysms: Passive Bystanders or Active Contributors?

**DOI:** 10.31083/RCM44035

**Published:** 2025-10-23

**Authors:** Andrey Suslov, Sergey Shemyakov, Ivan Chairkin, Ivan Milto, Olga Dondup, Tigran Lazaryan, Natalya Chairkina, Eduard Sheptak, Oleg Ustinov, Anton Postnov

**Affiliations:** ^1^Chazov National Medical Research Center of Cardiology, 121552 Moscow, Russia; ^2^Pirogov Russian National Research Medical University Moscow, 117513 Moscow, Russia; ^3^Petrovsky National Research Center of Surgery, 119991 Moscow, Russia; ^4^Siberian State Medical University (SSMU), 634050 Tomsk, Russia; ^5^Seversk Biophysical Research Centre (SBR Centre FMBA of Russia), 636013 Seversk, Russia

**Keywords:** thoracic aortic aneurysm, dissection, human herpesvirus, aortic morphology

## Abstract

The fundamental mechanism of thoracic aneurysm dissection involves morphological and functional reorganization of the aorta, accompanied by a reduction in the biomechanical parameters of the arterial wall. Functional zones with high receptor density are distinguished along the aorta. The autonomic reflex arc ensures the functional feasibility for a virus to penetrate the functional zones of the aorta. Numerous clinical and experimental studies demonstrated that necrotic changes specific to aneurysms develop in the middle sheath of the aorta. Therefore, necrosis of the aortic media may result from damage by the virus to the middle layer of the aorta. Further research should focus on the potential role of herpesviruses in medial vascular wall necrosis. The development of antiviral therapy for patients with aortic aneurysms will help stop medial necrosis in the aortic wall and possibly reduce mortality rates.

## 1. Introduction

Cardiovascular diseases (CVDs) are currently becoming increasingly relevant. Modern methods of diagnosis and prevention do not allow one to achieve acceptable treatment outcomes for this category of patients [1]. The morphological aspects of CVD pathogenesis, including the role of inflammation and the impact on tissue integrity, are crucial in understanding the underlying causes and progression of the disease [2]. Aortic aneurysm is the most common aortic disease. The overall incidence of thoracic aneurysm ranges from 5 to 10 cases per 100,000 population [3]. The high mortality is caused by aneurysm dissection and rupture. Approximately 20% of patients with thoracic aortic aneurysm dissection die before they develop noticeable symptoms or are diagnosed [4].

The anatomical parts of the aorta have a heterogeneous morphological structure and exhibit different functional characteristics. Such segmental heterogeneity of the aorta is required to ensure selective responses of the vessel to external and internal stimuli. The selective response and heterogeneity of the aorta structure cause specific changes in the vascular wall under pathological conditions. For example, it is well known that in patients with atherosclerosis, pathological changes are observed in the inner layer, while the middle layer of the aorta is affected in patients with aneurysms [5]. Aortic aneurysm is characterized by morphological and functional restructuring of the aorta, leading to changes in the biomechanical characteristics of the vascular wall. Aortic aneurysms develop because of the disruption of the normal structure of the blood vessel wall, manifesting itself as degeneration of the middle layer, thinned and fragmented outer and inner elastic membranes, and degenerative changes in collagen and elastic fibers [6].

The fundamental histopathological elements of thoracic aneurysm dissection are morphological and functional restructuring of the aorta, reducing the biomechanical characteristics of the vascular wall. As a result, the chain of cellular and molecular cascades develops in response to vessel wall dissection by the blood flow. That is, activation of the cellular and molecular pathways occurs secondarily, in response to structural, morphological, and functional transformations of the vascular wall [7].

Therefore, it seems quite reasonable to study the etiology and morphology of dissection and rupture of thoracic aneurysms. Current research focuses on specific pathophysiological mechanisms of the development of thoracic aneurysms. Studies are being conducted in the field of molecular and cellular biology of thoracic aneurysms. Genetic predisposition to developing aneurysms is being actively investigated [8]. Several studies describing clinical cases of viral infection in patients with aneurysms of the thoracic and abdominal aorta, as well as peripheral arteries, have been published [9, 10, 11]. For a viral infection present, our laboratory formulates a scientific hypothesis revealing the mechanisms of viral spread to the aortic wall. The essence of the hypothesis is as follows: viral infection localized in the aortic wall may trigger an inflammation and activate structural, morphological, and functional reorganizations in the vascular wall. For reviewing the proposed hypothesis, a search across publications was conducted based on analyzing articles containing the keywords or keyword strings “thoracic aortic aneurysm”, “dissection”, “human herpesvirus”, and “aortic morphology” in the PubMed and Scopus databases as of June 2025.

## 2. The Evolutionary and Epidemiological Prerequisites for the Etiological Role of Human Herpesviruses in Thoracic Aneurysms

Viral infections are a serious threat to public health, as confirmed by the COVID-19 pandemic. Viruses of the Herpesviridae family (more than 200 species) infect mammals, birds, reptiles, amphibians, fish, and bivalves. The members of the human herpesvirus family are widely distributed across the human population. It is believed that approximately 90–100% of the adult population is infected with at least one of the viruses belonging to the human herpesvirus family [12, 13]. According to the epidemiological data, the prevalence of cytomegalovirus (CMV) is 83% [14]; the prevalence of chickenpox in people aged 20–49 years is 98% [15]; according to various estimates, herpes simplex virus type 1 and 2 is present in 35–90% of the world’s population [16, 17, 18]; Epstein–Barr virus is detected in approximately 95% of people [19].

Herpesviruses are systemic, pantropic lymphoproliferative human immune pathogens having a prominent oncogenic potential, causing latent infection of cells and chronic inflammation [20, 21]. The mechanism of virus reactivation from the latent state has not been fully studied and may differ for different herpesviruses. The following factors are important in herpes infection reactivation: stressful situations, concomitant infectious diseases, injuries and surgical interventions, deficiency of micro- and macronutrients, hypovitaminosis, as well as ultraviolet and background radiation [22, 23].

A number of studies confirming the localization of herpesviruses in the nervous system have been conducted [24, 25]. The spread of herpesviruses into the nervous system through the vagus nerve, and virions were found in the sensitive ganglia of the spinal cord and aortic receptors in refs. [26, 27, 28].

In a study conducted in Sweden, involving 22 patients with abdominal aortic aneurysm, CMV infection was detected in almost all the samples in smooth myocytes of the wall of a pathologically altered vessel using the studied drugs with highly sensitive immunohistochemical staining, and Immunoglobulin G (IgG) was detected in the blood serum available from half of these patients [10].

Other researchers employed Polymerase Chain Reaction (PCR) methods (including quantitative real-time PCR) to show that CMV infection can stimulate local inflammation in the aorta, but is not the direct cause of most abdominal aortic aneurysms [29].

Using endothelial cell cultures or tissue endothelial preparations, a team of researchers found CMV-hybridizing endothelial cells in six out of eight virtually normal human aorta and in sixteen out of eighteen affected aorta, as well as infected cells localized in both the inner and middle layers of the vascular wall, and antigen-positive cells were visualized in normal areas of the pathologically altered aorta and in areas of lipid strips, except for atherosclerotic plaques of the same vessels [26].

There are very few research publications describing the role of viruses in aneurysm etiology. Several studies have reported cases of vasculopathy developing in patients with recurrent herpes infection because of herpesvirus penetration into the arterial wall through sensory ganglia, which induces inflammation and pathological vascular remodeling as well as may cause ischemic stroke, aneurysms, artery dissection, and peripheral vascular disease [30, 31]. Rabelo *et al*. [32] presented a case report of a patient with an intracranial aneurysm. DNA of the Epstein–Barr virus was detected in the aneurysm wall in this patient. The COVID-19 pandemic attests to the potential role of viruses in the etiopathogenesis of aneurysms. Potential mechanisms responsible for the impact of the virus on the integrity of vascular endothelium, aortic wall injury, and aortic dissection have been reported [33, 34].

## 3. The Significance of the Morphometric Parameters of the Aorta in the Context of Thoracic Aneurysm Pathogenesis

The aorta is an elastic artery consisting of three layers. The inner layer of the aorta (tunica intima) is well-developed. Its cross-section looks like a double-contour cord, forming prominent wave-like tortuosity. The inner layer of the vessel is formed by the endothelium with a basal membrane, a subendothelial layer, and an internal elastic membrane. The basal part of the endothelial cells forms numerous processes oriented towards the subendothelial layer. These processes extend to the middle layer of the aorta, where they form nexuses with smooth muscle cells (myoendothelial contacts) [35, 36]. The subendothelial layer consists of loose fibrous connective tissue containing precursor cells of smooth muscle cells. Single, longitudinally directed smooth muscle cells are also found here. Deeper than the subendothelial layer, on the border with the middle layer, lies the internal elastic membrane. It separates the inner layer of the aorta from the middle one. This membrane is made up of a dense plexus of thin elastic fibers forming the inner circular and outer longitudinal layers. The elastic fibers of the internal membrane provide mechanical strength to the inner layer of the aorta. The middle layer follows the internal elastic membrane; the border between the inner and middle layers of the aorta is unclear [37, 38].

The middle layer of the aorta (tunica media) is well-developed. It ensures the elasticity and strength of the vessel. The middle layer of the aorta is formed by alternating layers of fenestrated elastic membranes with elastic and collagen fibers, contains smooth muscle cells, and a large number of proteoglycans. Elastic and collagen fibers in the middle layer form complexes that look like spirals connecting the fenestrated elastic membranes. The smooth muscle cells located here have a spiral orientation, according to the direction of the elastic fibers. Fenestrated elastic membranes, together with collagen and elastic fibers and short spindle-shaped smooth muscle cells located between them, form lamellar units of the aorta [39, 40].

A lamellar unit is a structural and functional unit of the aorta, found in the aorta of all mammals. It is approximately 11 µm thick. The thickness and number of lamellar units depend on the patient’s age and aorta topography. On average, the media of the thoracic aorta contains approximately 40–60 lamellar units. The number of lamellar units decreases by half when proceeding from the ascending aorta to the abdominal aorta [41].

The space between the lamellar units and the myofibroblastic cells is filled with extracellular matrix (ECM) elements. The ECM of the aorta is composed of a rich set of structural elements. Along with elastic and collagen fibers, the ECM consists of proteoglycans, glycosaminoglycans, and adhesive glycoproteins. The ratio between elastic and collagen fibers varies in different parts of the aorta. Evaluation of the quantitative characteristics demonstrates that the ratio between elastic and collagen fibers is the highest in the ascending aorta. The number of elastic fibers decreases when proceeding from the proximal to the distal part of the aorta. The study showed a 58% decline in the ratio between elastic and collagen fibers in the aorta below the origin of renal arteries [42]. The content of elastic fibers in the aorta is age-related. The half-life of elastic fibers was found to be 75 years [43]. The amount of collagen fibers, in turn, increases along the entire length of the aorta [44, 45]. Several studies have reported that quantitative changes in collagen and elastic fibers may reduce the biomechanical strength of the vessel, followed by the development of an aortic aneurysm [46]. During left ventricular systole, elastic fibers act as a reservoir for blood volume and ensure equal load distribution along the entire aortic wall. Collagen fibers perceive most of the load at physiological and elevated pressures. The structural elements of the middle layer of the aorta, integrated, form a single elastic framework of the vessel. A fenestrated external elastic membrane is located at the border between the middle and outer layers [47, 48, 49].

The outer layer of the aorta (tunica externa) is made of loose connective tissue containing a large number of thick collagen fibers oriented in the longitudinal direction. A small quantity of elastic fibers is present. Smooth muscle cells are also found occasionally. In the outer sections, the adventitia becomes looser. Nerve fibers innervating the aortic wall are found here, together with the vessels feeding the artery walls.

### Morphofunctional Features of the Aortic Receptor Zones

Despite receptor distribution throughout the aorta, the vessel has areas with the greatest accumulation of sensory nerves. Such receptor zones have a specific localization in the aorta. A number of the most clearly formed receptor zones can be identified today. The first zone is located on the ventral surface of the brachiocephalic trunk at the site when it departs from the aorta and involves the areas of vessel branching; the second zone resides on the ventral surface of the beginning of the left subclavian artery; the third one, on the ventral surface of the aortic arch; and the fourth zone is located in the area of the arterial ligament [50, 51]. Sensory (afferent) receptor zones of the aorta are located mainly in the middle layer, while being partially represented in the inner and outer layers of the aorta.

## 4. Morphofunctional Substantiation of the Pathway of Herpesvirus Entry Into the Aortic Wall

Neurogenic regulation is the key control mechanism of the vascular system. The regulation of vascular system activity is ensured by several ways of receiving information to control centers and a set of relatively independent executive mechanisms. The nervous system departments responsible for regulating blood circulation are represented at different levels of its organization, starting from the spinal level and followed by the brain stem, subcortical, and cortical centers of the nervous system [52, 53]. Neurogenic regulation of the aorta is among the key ways to control blood circulation in the body [54, 55].

The autonomic reflex arc is a morphofunctional unit ensuring neurogenic regulation of the aorta [56, 57]. The receptor zones of the aorta are the initial elements of the autonomic reflex arc. The sensory fibers coming from the receptors form a separate nerve (depressor nerve, or aortic nerve). This aortic nerve, as part of the vagus nerve, reaches the brain stem, where it forms synapses with neurons of the nucleus of a single pathway.

Aorta innervation is composed of nerve fibers of the aortic nerve, sensory fibers of the spinal nerves of the cervical and upper thoracic parts of the spinal cord, as well as postganglionic sympathetic fibers from the thoracic nodes of the sympathetic trunk. These structures form the thoracic aortic plexus surrounding the aorta.

**Fig. 1.**
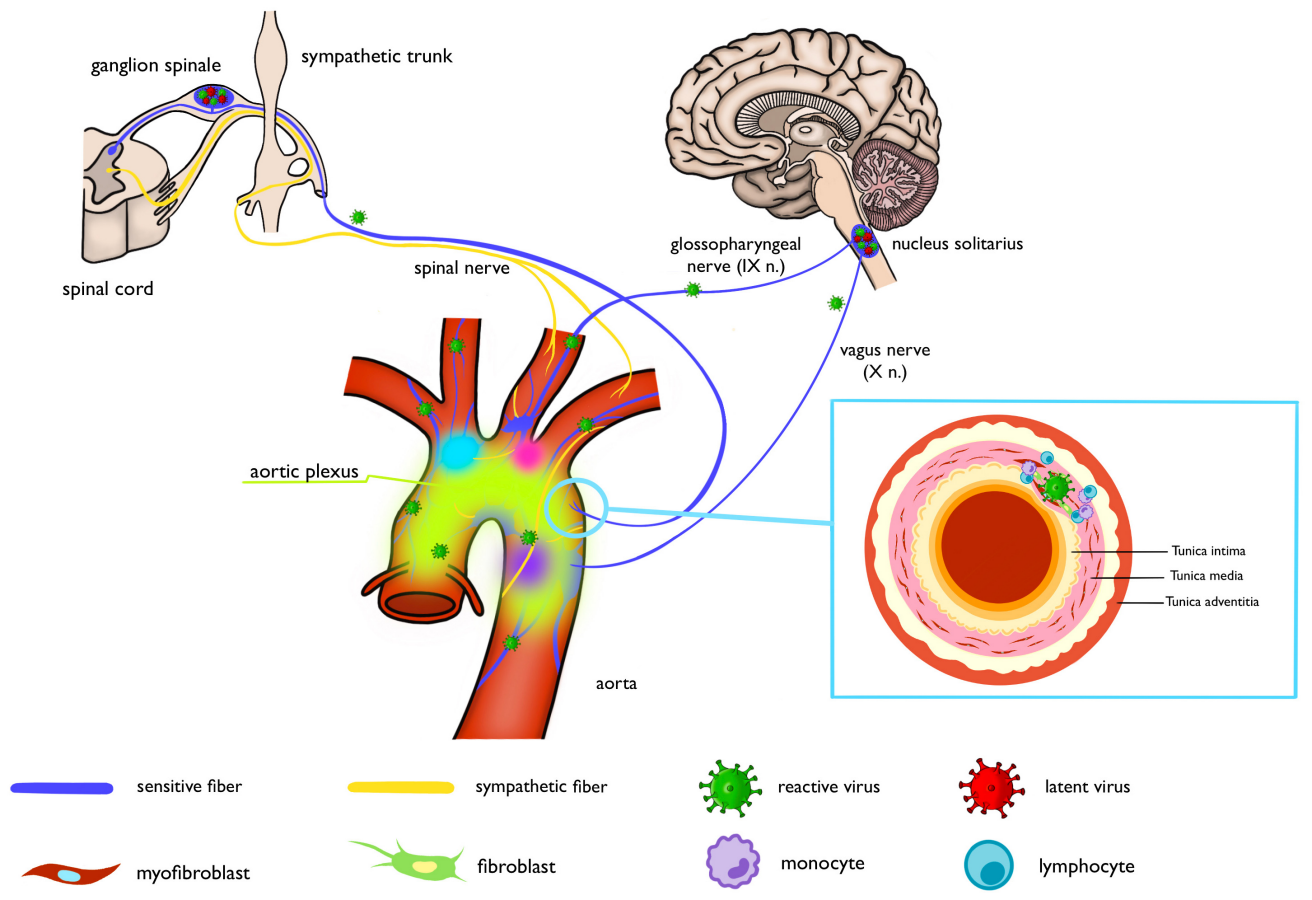
**Schematic of the proposed viral entry route**. Created with Procreate, Savage Interactive Pty Ltd, Australia.

The studies demonstrating herpesvirus spread along the nervous system via the vagus nerve, as well as the discovery of viral virions in sensory ganglia of the spinal cord and aortic receptors, suggest that the virus spreads from the nervous system into the aortic wall through nerve fibers of the aortic plexus (Fig. 1).

## 5. Conclusions

The literature analysis data allow one to identify several functional zones of the aorta. Such functional zones are topographically located on the ventral surface of the aortic arch, in the area of origin of the brachiocephalic trunk, the left subclavian artery, and within the arterial ligament. Meanwhile, the morphological features of the respective aortic segments determine the distinctive properties of the biomechanical parameters of the vessel.

A viral infection localized within the functional zones of the aorta can elicit an inflammatory response and potentially activate the structural and functional transformation of aortic aneurysms. The following arguments are provided as evidence:

- Taking into account the spread of herpesviruses among humans, it is logical to assume that patients with thoracic aneurysms are also infected with viruses belonging to this family. A characteristic feature of these viruses is their tropism to many types of cells, including cells of the vascular wall of the aorta;

- Morphological data are currently available, indicating that human herpesvirus infect cells of the central and peripheral nervous system;

- The receptor zones of the aorta contain a large number of receptors, enabling the herpesviruses to spread through sensory fibers into the vessel wall. The receptor zones of the aorta correspond to the aorta topography, where the aneurysm sites are located;

- Most sensory receptors reside in the middle membrane of the aorta. Numerous clinical and experimental studies have proved that aneurysm-specific necrotic changes develop in the middle tunic of the aorta. It is possible that necrosis of the aortic media results from damage to the middle aortic membrane caused by the virus.

However, the role of viral infection as an activator of tissue changes can be short-term; i.e., the virus only triggers the aortic inflammation mechanisms, and subsequent vascular transformation, including blood vessel rupture, develops under the influence of other factors. After activating inflammation in the vessel wall, the virus becomes inactive and resides only in the sensory ganglia of the nervous system. For example, a PCR study of thoracic aortic aneurysm samples aiming to detect Varicella Zoster virus revealed no viral DNA in the vessel wall [58].

## 6. Future Prospects

Further research should focus on the possible role of herpes family viruses in the development of medial vascular wall necrosis. The advancement of antiviral therapy for patients with aortic aneurysms will help stop medial necrosis in the aortic wall and possibly reduce the number of deaths. Currently, there are insufficient data to justify antiviral therapy for everyone or even for patients at risk of developing an aneurysm; however, the problem discussed in this review is a highly relevant medical and social aspect for further research. Theoretical and clinical studies are needed to investigate the pathogenetic mechanisms of aortic aneurysm and dissection development, as well as to improve diagnostic algorithms in this cohort of patients, including additional examination for herpesvirus infection. The diagnostics of herpesvirus infection seems necessary for all patients with thoracic aortic aneurysm. However, its clinical significance is appropriate only when considering the stage of morphological transformation of the vascular wall. First of all, the diagnostics of herpesvirus infection should include a group of patients with aneurysm dissection, without vascular wall rupture. If a viral infection is detected in such patients at the prehospital stage or in the preoperative period, antiviral therapy will be indicated, aiming to prevent inflammation activation in the aneurysm wall and, accordingly, inhibit further pathological transformation of the vascular wall, which will allow surgeries to be carried out on a scheduled basis. The reviewed evidence is equally suitable for both syndromic and sporadic aneurysms, which underscores the universality of our hypothesis.
